# Evaluation of Generative Artificial Intelligence Implementation Impacts in Social and Health Care Language Translation: Mixed Methods Case Study

**DOI:** 10.2196/73658

**Published:** 2025-09-17

**Authors:** Miia Martikainen, Kari Smolander, Johan Sanmark, Enni Sanmark

**Affiliations:** 1 Department of Software Engineering, School of Engineering, Lappeenranta-Lahti University of Technology, Yliopistonkatu 34, Lappeenranta, 53850, Finland, 358 449014240; 2 Department of Research, Development and Innovation, Western Uusimaa Wellbeing Services County, Espoo, Finland; 3 Department of Medicine, Faculty of Medicine, HUS Helsinki University Hospital and University of Helsinki, Helsinki, Finland

**Keywords:** generative artificial intelligence, large language model, ChatGPT, pretrained language model, language translation, machine translation evaluation, public social and health care

## Abstract

**Background:**

Generative artificial intelligence (GAI) is expected to enhance the productivity of the public social and health care sector while maintaining, at minimum, current standards of quality and user experience. However, empirical evidence on GAI impacts in practical, real-life settings remains limited.

**Objective:**

This study investigates productivity, machine translation quality, and user experience impacts of the GPT-4 language model in an in-house language translation services team of a large well-being services county in Finland.

**Methods:**

A mixed methods study was conducted with 4 in-house translators between March and June 2024. Quantitative data of 908 translation segments were collected in real-life conditions using the computer-assisted language translation software Trados (RWS) to assess productivity differences between machine and human translation. Quality was measured using 4 automatic metrics (human-targeted translation edit rate, Bilingual Evaluation Understudy, Metric for Evaluation of Translation With Explicit Ordering, and Character n-gram F-score) applied to 1373 GAI-human segment pairs. User experience was investigated through 5 semistructured interviews, including the team supervisor.

**Results:**

The findings indicate that, on average, postediting machine translation is 14% faster than translating texts from scratch (2.75 vs 2.40 characters per second, *P*=.03), and up to 37% faster when the number of segments is equalized across translators. However, productivity varied notably between individuals, with improvements ranging from −2% to 102%. Regarding translation quality, 11% (141/1261) of Finnish-Swedish and 16% (18/112) of Finnish-English GAI outputs were accepted without edits. Average human-targeted translation edit rate scores were 55 (Swedish) and 46 (English), indicating that approximately half of the words required editing. Bilingual Evaluation Understudy scores averaged 43 for Swedish and 38 for English, suggesting good translation quality. Metric for Evaluation of Translation With Explicit Ordering and Character n-gram F-scores reached 63 and 68 for Swedish and 59 and 57 for English, respectively. All metrics have been converted to an equivalent scale from 0 to 100, with 100 reflecting a perfect match. Interviewed translators expressed mixed reviews on productivity gains but generally perceived value in using GAI, especially for repetitive, generic content. Identified challenges included inconsistent or incorrect terminology, lack of document-level context, and limited system customization.

**Conclusions:**

Based on this case study, GPT-4–based GAI shows measurable potential to enhance translation productivity and quality within an in-house translation team in the public social and health care sector. However, its effectiveness appears to be influenced by factors, such as translator postediting skills, workflow design, and organizational readiness. These findings suggest that, in similar contexts, public social and health care organizations could benefit from investing in translator training, optimizing technical integration, redesigning workflows, and implementing effective change management. Future research should examine larger translator teams to assess the generalizability of these results and further explore how translation quality and user experience can be improved through domain-specific customization.

## Introduction

Generative artificial intelligence (GAI) is being explored as one prospective innovation to address the pressing economic sustainability challenges of the Finnish public social and health care sector. GAI refers to digital technologies capable of producing original content, such as text, images, audio, and video by learning semantic patterns from vast amounts of pretraining data [1,2]. In the context of this study, GAI specifically refers to a large language model (LLM) engine called GPT-4 developed by a research organization called OpenAI.

In 2024, the Finnish Ministry of Social Affairs and Health established an artificial intelligence (AI) ecosystem for social and health care services to enhance care quality, service delivery productivity, and economic growth through AI-driven innovation [3]. Despite the growing strategic focus on GAI, operational AI maturity of the public sector remains at an early development stage [4,5]. Hjaltalin and Sigurdarson [6] consolidated 28 governmental AI strategies, including Finland’s, into three national AI discourses: (1) empowerment through information, (2) enhanced administrative practices, and (3) improved service delivery. Implementing GAI in administrative practices presents a significantly lower barrier to entry due to lower compliance risk, as secondary use of social and health care data and application of GAI in service delivery are subject to stringent regulation. For instance, the European Union regulatory frameworks of the AI Act and the General Data Protection Regulation (GDPR) have a significant impact on the usage of GAI in public administration.

In Finland, 21 well-being services counties, alongside 3 other governance entities (HUS Group, the joint authority for Helsinki and Uusimaa), are responsible for public social, health, and rescue services [7]. Approximately 30% of these entities are legally required to provide services in both national languages, such as Finnish and Swedish [8]. The case organization of this study is Western Uusimaa, the third largest well-being services county in Finland. Western Uusimaa Wellbeing Services County (Länsi-Uudenmaan hyvinvointialue, LUVN) is not only a bilingual region but also a region where population growth is projected to be driven primarily by an increase in residents whose native language is neither Finnish nor Swedish [9]. A similar trend can be observed globally due to globalization, while the legislation of many countries still requires services to be provided in the official languages of the country. Translation capability is crucial in health care settings, because language barriers can lead to issues in patient safety and outcomes [10]. Consequently, language translation services are becoming an increasingly critical support function across the health care sector.

Machines have been used to support language translation from as early as the 1950s [11], but the more sophisticated, machine learning–based technologies have been available from approximately 2015 onwards [12]. Language translation is among the most widely recognized applications of LLMs in the health care sector [13]. Despite considerable technological progress in recent years, the quality of machine translation (MT) has not yet reached the level of experienced human translators [14,15]. Thus, postediting is an essential practice of modern translation work [16].

Earlier reviews, such as Wang et al [16], examined studies conducted between 2007 and 2011 and reported conflicting results regarding productivity gains associated with prior-generation MT. Several studies reported improvement in translator productivity, while others have found no significant increase or even a slight speed reduction. A study by Macken et al [14] found a 14% speed improvement with neural MT for the English-Finnish language pair and a 12% speed increase with statistical MT for English-French in real-world settings. A more recent study by Merali [17] evaluates the productivity effect of LLMs. The study found that a 10-fold increase in model training compute led to significant improvement in task completion speed (12.3% relative to baseline and grades by 0.18 SDs), translation quality, and translator earnings. Notably, the productivity benefits were reported higher among less experienced translators.

GAI is fundamentally transforming language translation. However, there is currently limited evidence on real-life impacts of GAI-based MT, particularly regarding specialized sectors, such as social and health care. This study aims to investigate the productivity impact, translation quality, and user experience of a GAI-based plugin for computer-assisted translation (CAT) software in social and health care services.

## Methods

### Overview

Language translation services of the case organization LUVN are coordinated and produced by an organizationally centralized in-house team of 4 translators. Translators primarily translate from Finnish to Swedish. A minor portion of translation tasks involves the language pairs, which are Swedish-Finnish and Finnish-English. Translators have translation experience ranging from 1 to 13 years and industry-specific experience of 0‐2 years in social and health care sectors. Translators’ educational backgrounds focus on literature, translation, and social sciences, but every one of them does have prior experience with MT aids.

The in-house translation department did not have a standardized practice of using MT aid prior to the GAI-based plugin. The GAI-based plugin was used independently and was not combined with any other MT plugins. The study was conducted during the early adoption stage, and no term bases were used for the GAI-based plugin. In addition, only the system prompt was used. The translators neither have visibility to the defined system prompt nor do they use any task-specific prompts.

Mixed methods were chosen to capture both objective and experiential aspects of GAI adoption. The quantitative and qualitative components were collected sequentially but analyzed in parallel to support triangulation of findings. Data collection was conducted from March until June 2024 and measured 3 dimensions, such as productivity, quality, and user experience. Translators processed usual translation materials, excluding any materials with sensitive or personal data.

### Productivity

The productivity of the language translation was assessed as translation speed according to the earlier studies [18]. The data were extracted from the files in the SDL XML-based localization interchange file format in that Trados software (Trados Studio 2022, version SR1 - 17.1.6.16252, RWS Group), produced for each translation project. Translators were instructed to translate each text so that half of the segments are translated from scratch and the other half using GAI-based MT (AI Professional Plugin for Trados, RWS Group). The plugin is using the Microsoft Azure, OpenAI GPT-4 language model service. To reduce bias, translators were advised to randomly select each time whether they start with or without GAI. Translators retrieved GAI-based MT at a segment level only for segments without translation memory (TM) matches. Translators collected 66 documents in total, including 2946 segments. The data were filtered with four criteria:

1. Comparison between translation from scratch and postediting: Segments from TM (708), from other automated translations, such as auto-propagation and direct copies from source segments (257), as well as segments that were postedited based on the aforementioned other automated translation outputs (332) were excluded.
2. Translation time calculation: Trados does not record translation time directly, but 2 time stamps “Creation_date_and_time” and “Modified_on” were used to estimate translation time by calculating the time difference between the time stamps of consecutive segments. Only positive translation times were accepted. A negative time stamp is primarily caused by a translator revisiting the segment at a later stage, resulting in nonconsecutive segments.
3. Translation time maximum threshold: Segments with a translation time of more than 30 seconds per word were excluded, as this suggests the translator was not actively engaged in the task. This assumption is aligned with prior research [19].
4. Translation time minimum threshold: Segments with translation times under 0.5 seconds per word were excluded, as such results likely indicate typographical errors or software malfunctions, making the data unreliable. This assumption is aligned with prior research [19].

The final dataset for comparative analysis consisted of 908 segments. Of these, 47% (n=427) of segments were postedited while 53% (n=481) of segments were translated from scratch (Table 1). A total of 62% (427/692) of the GAI postedited segments and 50% (481/957) of the segments translated from scratch were incorporated into the analysis. The segments included in the analysis contained a higher average number of words and characters compared to those excluded.

**Table 1.** Summary of language translation segment data from an in-house translation team of a Finnish well-being services county.

Measure		Segments excluded from the analysis				Segments included in the analysis		
		Postediting GAI^a^ output^b^	Human translation^b^	Other^c^	Total	Postediting GAI output	Human translation	Total
Number of segments		265	476	1297	2038	427	481	908
Number of words in a segment, mean (SD)		7.8 (5.9)	7.9 (5.5)	—^d^	—	9.7 (5.4)	8.9 (5.2)	9.3 (5.3)

a GAI: generative artificial intelligence.

b Segments excluded based on filtering criteria 2‐4.

c Segments excluded based on filtering criterion 1.

d Not applicable.

### Quality

Target quality is defined as quality accepted by a human translator [14,20,21]. The translation process began with the pretranslation of the entire text using GAI, incorporating TM. The file in the SDL XML-based localization interchange file format containing the machine-generated translations was saved, after which the translators performed postedits and saved the modified file under a different name. Differences between the original GAI output and the final human-reviewed translation were then compared. Similar to the collection of productivity data, quality data were collected during the translators’ regular workflow.

Although data collection was initiated with 4 translators, only 3 provided datasets that could be included in the quality analysis. For 1 participant, no analyzable data were recorded due to a technical issue during the data capture process. Despite prior instructions and support, the system did not retain segment-level data for this individual, resulting in an empty export file. Consequently, their data could not be used in the final analysis.

The final dataset included 47 documents and 1766 segment pairs. TM was used for 20% (353/1766) of segments, with most matches being exact (254/353). To refine the dataset, TM segments and other automatically generated segments were excluded, resulting in a final dataset of 1373 segment pairs for analysis. Each segment pair consisted of a raw GAI output and its postedited version corresponding to a single source language segment.

The quality of MT was estimated using 4 established and widely adopted automatic metrics: human-targeted translation edit rate (HTER)–score, Bilingual Evaluation Understudy (BLEU) score, Metric for Evaluation of Translation With Explicit Ordering (METEOR)–score, and character-level metric Character n-gram F-score (ChrF). HTER aligns well with real-world use cases, as it evaluates the effort required to postedit MT output into an acceptable human translation, but it only calculates the minimum amount of effort to finalize translation [14]. More sophisticated automatic measures BLEU and METEOR are both widely used methods for evaluating MT quality [22]. A BLEU score above 40 indicates high-quality translations while scores between 30 and 40 reflect translations that range from understandable to good [23]. Finnish is a morphologically rich language, which means it has a complex system of word formation and inflections. ChrF performs particularly well for such languages [24].

### User Experience

A total of five 60-minute videoconference interviews were conducted in May 2024. The sessions were recorded and automatically transcribed for analysis. The aim was to explore the impact of GAI implementation on translation services, focusing on the perceived productivity, MT quality, and user experience. The interview questions are listed in the Multimedia Appendix 1.

### Ethical Considerations

This study was reviewed and approved by Western Uusimaa Wellbeing Services County on March 6, 2024 (approval number 636/13.01.00/2024). All participants were informed about the purpose of the study and provided written informed consent prior to participation. Recruitment of participants was facilitated through managerial channels. All 4 in-house translators were invited to participate, and participation was voluntary. After initial coordination with the management, the researcher maintained regular contact with the in-house translators to (1) provide study information, (2) support data collection, and (3) address any questions related to the research process. The consent process included permission for collection, analysis, and publication of data in anonymized form. All analyzed and reported data were processed to ensure anonymization. No identifiable information is included in the published results or supplementary materials. Participants were not provided with monetary or material compensation, as their involvement in the study took place during regular work hours as part of their normal professional duties.

## Results

### Productivity

Human translation speed was 2.40 characters per second (total of 481 segments) and postediting a GAI output speed was 2.75 characters per second (total of 427 segments). Hence, the postediting GAI-generated segment was, on average, 14% faster than translating from scratch (characters per second). Due to the nonnormal distribution of the dataset, the Mann-Whitney *U* test was applied, indicating a statistically significant difference (*P*=.03).

Significant variation occurs among translators in terms of productivity differences and the volume of segments generated (Table 2). Translator A produced 61% (555/908) of the total segments, with a minor decrease in productivity when using GAI. In contrast, the productivity of the other 3 translators increased by 18%, 28%, and 102%, respectively, when using GAI. To provide additional context to the observed variation in productivity, a supplementary analysis was conducted in which the translated segments were evenly distributed across translators. While this approach is not a standard practice, the analysis is included as a complementary perspective to illustrate how differences in workload distribution may influence the overall productivity outcomes. When calculating productivity differences by equally distributing segments across translators, postediting GAI-generated output is, on average, 37% faster than translating from scratch.

**Table 2.** Comparative analysis of translation speed in a case study of an in-house translation team within a Finnish well-being services county. Segments are analyzed by translator.

Measure		Translator			
		A	B	C	D
Segments, n (%)		555 (61)	207 (23)	102 (11)	44 (5)
Average translation speed (characters per second)					
Human translation		2.4	1.7	3.5	2.2
Postediting GAI^a^ output		2.3	2.1	7.2	2.6
Difference (%)		–2	28	102	18

a GAI: generative artificial intelligence.

### Quality

In the context of this study, maximum quality is defined as MT output that requires no postediting by a human translator. Notably, 141/1261 (11%) Finnish-Swedish segment pairs were accepted by translators without edits, whereas 18/112 (16%) Finnish-English segment pairs required no human intervention. All other target segments underwent postediting by human translators. The quality of MT was evaluated using 4 automatic metrics (Table 3).

Based on the average BLEU scores, the MT output for English achieved a good quality level while the output for Swedish was of high quality. However, HTER scores indicate room for quality improvement, and scores of 55 for Swedish and 46 for English suggest that approximately half of the words required postediting. It is important to note, however, that “edit” does not necessarily imply that half of the words were incorrect; edits may involve insertions, deletions, substitutions, or shifts.

**Table 3.** Automatic quality metrics for GAI-based^a^ machine translation output in a case study of an in-house translation team in a Finnish wellbeing services county.

Measure		Target segment language	
		Swedish	English
Total number of segments, n		1261	112
GAI output accepted without edits			
Segments, n (%)		141 (11)	18 (16)
Machine translation output quality metric (average), mean^b^,^c^			
HTER^d^,^e^		55	46
BLEU^f^		43	38
METEOR^g^^h^		63	59
ChrF^i^		68	57

a GAI: generative artificial intelligence.

b Scale 0-100, where 100=perfect match.

c The reported HTER, BLEU, METEOR, and ChrF values are aggregate machine translation evaluation metrics calculated over the entire dataset. Therefore, a standard deviation is not applicable in this context.

d HTER: human-targeted translation edit rate.

e Multiplied by 100, inverted and negative values changed to 0 to convert the metric to equivalent scale from 0 to 100.

f BLEU: Bilingual Evaluation Understudy.

g METEOR: Metric for Evaluation of Translation With Explicit Ordering.

h Multiplied by 100 to convert the metric to equivalent scale from 0 to 100.

i ChrF: Character n-gram F-score.

### User Experience

Interviews revealed diverse user experiences on the impact of GAI. Qualitative findings supported the contextualization of the variability observed in quantitative productivity measures. One translator assessed that GAI accelerates their overall translation productivity, while another translator reported that it actually slowed down their work due to quality deficiencies and workflow inefficiencies. Two translators perceived the overall impact as neutral, with productivity varying based on text type, complexity, and terminology. Overall, 3 out of 4 translators expressed a desire to continue using a similar GAI tool in the future.

The translators acknowledged the potential of GAI to improve translation quality but noted its current limitations, including inconsistent outputs and challenges with industry-specific terminology, gender, and concord issues. In addition, variations in translation quality, even with identical prompts, and a lack of document-level context were identified as significant challenges. Despite these shortcomings, translators found that GAI is effective in improving clarity of vaguely written source texts and for longer, repetitive texts with generic terminology.

Suggestions for improving GAI included refining workflows within CAT tools, improving terminology management, and using context-specific fine-tuning. Translators expressed cautious optimism about GAI’s potential to enhance both productivity and translation quality but emphasized the need for tailored development and ongoing support to address its limitations.

## Discussion

### Principal Findings

In this study, the impacts of implementing a GAI-based MT solution on productivity, quality, and user experience within the in-house language translation services of a large Finnish well-being services county were assessed. Findings from this case study indicate potential productivity benefits of GAI in public sector translation flows. Specifically, postediting GAI-generated outputs was on average 14% faster than translating segments from scratch.

Although GAI has already improved the productivity of translators in the case organization, the potential of GAI remains partially unrealized. Further productivity gains are more likely to be harnessed by prioritizing the enhancement of organizational GAI capabilities, including the development of human skills, context-specific configuration, and fine-tuning of GAI systems, as well as effective change management, rather than solely emphasizing technological development. In addition, the integration of GAI tools in social and health care requires addressing ethical and societal questions, particularly given the sensitive nature of information handled and potential consequences of mistranslations.

The results are consistent with the study conducted by Macken et al [14] that similarly reported a 14% increase in translation speed when using neural MT. The efficiency of postediting process is significantly influenced by output quality, source text complexity, and translators’ technical ability [25,26]. The proportion of 100% accurate MT segments in Finnish-Swedish language pairs was merely 11%, highlighting the need for improving the quality of GAI MT quality to enhance productivity. It is also important to note that postediting involves distinct skills compared to traditional translation [27,28]. For instance, translators may choose to integrate their stylistic preferences into the text rather than limiting the effort to minimal corrections, which affects overall productivity.

The automatic metrics calculated in the study indicate that the quality of MTs can still be significantly improved. For instance, HTER scores of 55 and 46 (Swedish and English, respectively) suggest that a postedit is needed for approximately every other word in a segment to finalize MT output. In the study, translators frequently encountered issues with industry- and organization-specific terminology, which mirrors findings by Turner et al [29] and Zappatore and Ruggieri [30]. They identified domain adaptation as a critical challenge for MT adoption, particularly in specialized sectors, such as health care. Another significant barrier to GAI adoption was the current integration with CAT software (Trados), which operates at the segment level rather than incorporating the broader document-level context. This limitation undermines one of the core strengths of GPT-based models, which are designed to account for long-range dependencies and contextual information [31].

Prior research on MT user experience remains very limited. The primary focus of the earlier research has been on the technical development of MT, with less emphasis on ensuring the usefulness and usability of MT tools for translation professionals [18]. In general, translators tend to dislike postediting and prefer translation from scratch, even if MT post-editing is faster [32]. This observation is based on statistical MT and neural MT aids and may not fully reflect translation technology preferences regarding more recent LLM-based solutions. Recent studies have highlighted that professional users express concerns regarding the quality and reliability of ChatGPT when used for translation tasks. However, they also recognize the potential of such tools, particularly for auxiliary tasks, such as drafting, summarizing, or generating translation suggestions [33,34]. In addition, translators with prior experience in postediting exhibited more favorable attitudes toward the method [26].

In this case study, translators generally expressed a positive attitude toward GAI, with most preferring its use in suitable contexts over traditional methods. Translators’ own assessments on productivity impacts of the GAI solution in use were more conservative than the results of the quantitative analysis.

Beyond technical challenges, human and organizational factors seem to play a critical role in realizing the full potential of GAI. The transition from conventional translation to MT postediting redefines the role of the human translator, shifting it from producing and crafting text to reviewing and refining output [25]. Translators emphasized the need for stronger technical support and adequate time for skill development, underscoring that effective adoption requires both training and hands-on experience with the tool. Negative experiences early in the adoption process, exacerbated by high workloads as well as inadequate preparation and resourcing, illustrate the importance of effective change management when introducing new technologies. These findings are consistent with Mikalef et al [5], who emphasize the importance of basic resources and human skills in AI adoption, alongside technological infrastructure and data capabilities.

Translators acknowledged the future potential of GAI systems to deliver faster and more consistent translations, reduce reliance on outsourcing, and improve workflow efficiency. However, concerns regarding job security and the loss of personal translation styles underscore the need for a balanced approach that leverages GAI as a tool to augment, rather than replace, human expertise. As Sezgin [35] and Yang et al [36] argue, the goal of GAI in fields such as public social and health care should be to support human professionals, not to automate all tasks, given the critical and complex nature of the work involved.

### Strengths and Limitations

This mixed methods case study combines quantitative and qualitative methods to holistically assess impacts of GAI in language translation services within a public social and health care organization. It is based on widely used metrics and research design from previous studies. Another strength of the study is the practical relevance as it reflects real-life conditions to measure impacts of GAI in the specialized domain of social and health care.

Given the limited sample size, the findings need to be interpreted with caution and are not directly generalizable. In general, language translation teams within public social and health care sectors are small, which limits the sample size. The primary aim of the study is to explore early-stage implementation and practical implications of a GAI tool in a real-world setting. While this approach limits statistical generalization potential, investigating actual deployment in an authentic work environment can offer valuable, practice-oriented insights in how such tools are adopted, used, and experienced by professional translators.

In addition, the study likely captures only part of GAI’s potential, as its effectiveness is influenced by the organization’s current capability to integrate and optimize the technology. The data derived from the CAT software Trados also present limitations in fully representing the complexity of translators’ daily workflows. For instance, segment-level time stamps in Trados may not accurately account for time spent on tasks due to interruptions or unforeseen delays. As a result, several assumptions were made during data collection, analysis, and interpretation, as outlined in detail in the “Methods” description.

Although well-established and widely adopted automatic evaluation metrics (BLEU, METEOR, ChrF, and HTER) were applied in this study, they have known limitations particularly in capturing semantic adequacy and fluency. More recent neural-based metrics, such as Crosslingual Optimized Metric for Evaluation of Translation [37] and Bilingual Evaluation Understudy with Representations from Transformers [38] have been proposed to better correlate with human judgment. Although these metrics were not widely standardized at the time of conducting this study, their growing potential should be considered for future research.

### Conclusion

This mixed methods study demonstrates promising productivity potential of using GAI in language translation within public social and health care sector. While quantitative data captured measurable productivity gains from postediting GAI outputs, translators expressed more reserved views on its overall impact, suggesting a gap between observed efficiency and perceived benefit. Realizing the potential of GAI depends not only on technological performance but also on organizational capability. Based on the insights gathered in this study, several practical strategies are suggested to support effective GAI adoption in public social and health care sector:

- Invest in upskilling: Provide training and technical support to help translators effectively postedit and work with GAI outputs.
- Redesign operational workflows: Collaborate with translators to update translation workflows for effective integration of GAI technology, and create best practices for, for example, prompt formulation.
- Optimize GAI performance: Continuously refine GAI systems to improve translation quality, which in turn enhances productivity and user experience.
- Address ethical considerations: Recognize and mitigate translators’ concerns related to new technology. Ensure human oversight to secure quality control, especially in sensitive domains such as social and health care.
- Monitor and evaluate impact: Define clear operational objectives. Regularly assess the effectiveness of GAI tools in translation services to support continuous development and informed decision-making.

Future research should aim to expand the scope of investigation to include larger-scale studies, providing more robust evidence on the impacts of GAI. In addition, greater academic attention should focus on end-user perspectives and evaluations in real-world settings. Further exploration of the specific components of GAI capability and their influence on productivity, quality, and user experience could offer valuable guidance for optimizing the design, implementation, and operational use of GAI systems in health and social services sector.

## Supplementary material

10.2196/73658 Multimedia Appendix 1 Interview questions.

## Abbreviations

AI: artificial intelligence
BLEU: Bilingual Evaluation Understudy
CAT: computer-assisted translation
ChrF: Character n-gram F-score
GAI: generative artificial intelligence
GDPR: General Data Protection Regulation
HTER: human-targeted translation edit rate
LLM: large language model
LUVN: Länsi-Uudenmaan hyvinvointialue
METEOR: Metric for Evaluation of Translation With Explicit Ordering
MT: machine translation
TM: translation memory
