# Supplementary material for: Evaluation of Generative Artificial Intelligence Implementation Impacts in Social and Health Care Language Translation: Mixed Methods Case Study
Source: JMIR Form Res. 2025 Sep 17;9:e73658. doi: 10.2196/73658 (PMC12443352; doi:10.2196/73658)
Supplement: Multimedia Appendix 1 [file formative-v9-e73658-s001.docx]

| **#** | **Interview question** | **Question type** |
| --- | --- | --- |
| 1 | What is your job role in the wellbeing services county? | Open |
| 2 | Which language pairs do you translate? | Open |
| 3 | How long have you worked as a professional translator? | Open |
| 4 | How long have you worked as a professional translator in public social and healthcare sector? | Open |
| 5 | Have you used AI-assisted machine translation before? How long? | Yes/no; open |
| 6 | How would you assess the translation speed with the two different methods being compared – GAI output post-editing and translating from scratch? | Open and options: Do you work   - slower, - at the same pace, - or faster |
| 7 | How would you assess the quality of GAI-assisted machine translation considering the requirements of the public social and healthcare sector? If you identify any quality issues, could you describe them in more detail? | Open |
| 8 | How would you rate the average quality of GAI-assisted machine translation? | Open and options:   - Excellent - Good - Satisfactory - Poor |
| 9 | Do you prefer to work without GAI-assisted machine translation or with its support? Why? | Yes/No; Open |
| 10 | Do your preferences differ depending on the type of material being translated? If yes, in what way? | Yes/No; Open |
| 11 | In your opinion, what are the key opportunities for utilizing GAI in the public social and healthcare sector from the perspective of a translation professional? | Open |
| 12 | In your opinion, what are the main challenges or issues in utilizing GAI in the public social and healthcare sector from the perspective of a translation professional? | Open |
| 13 | How would you improve the use of GAI-assisted machine translation in LUVN? | Open |
| 14 | What kind of general ideas do you have for developing the language translation services team operations in LUVN? | Open |
